# Exercise Habits and Preferences of Community-Dwelling Older Adults with Chronic Pain: An Exploratory Study

**DOI:** 10.3390/healthcare13040384

**Published:** 2025-02-11

**Authors:** Ziji Chen, Mimi Mun Yee Tse, Bonny Yee Man Wong

**Affiliations:** School of Nursing and Health Sciences, Hong Kong Metropolitan University, Hong Kong, China; s1348285@live.hkmu.edu.hk (Z.C.); bymwong@hkmu.edu.hk (B.Y.M.W.)

**Keywords:** aged, chronic pain, community health, exercise, health behavior

## Abstract

Introduction: This study explored the exercise habits of community-dwelling older adults with chronic pain, examining the relationship between pain, physical activity, daily life impacts, and psychological effects. Method: The study was conducted through a cross-sectional approach and semi-structured interviews with five participants aged fifty and above. Result: The findings revealed that exercise participation among those with chronic pain was significantly lower than in the non-pain participants, particularly for those exercising more than three times weekly (*p* = 0.012). Hypertension (59.64%) and arthritis (39.32%) were common among the respondents. Pain was predominantly reported in the lower back, legs, shoulders, and arms, severely affecting quality of life. Additionally, anxiety and depression were increasingly prevalent in this population, presenting greater challenges than financial constraints or lack of motivation. Lower impact exercises like walking were more doable, and social support and a good environment increased exercise engagement. Conclusions: We determined that interventions for older adults with chronic pain should address both physiological and psychological factors to boost exercise participation. This research emphasizes feasible exercise types and key factors to enhance engagement. Future research should focus on developing targeted intervention programs that incorporate these findings to improve the quality of life for this population.

## 1. Background

Global aging presents a universal challenge, as advancements in economic and medical technologies have enhanced life expectancy substantially. According to WHO estimates, by 2050, the proportion of the global population aged 65 and over is expected to rise to 16% from 10% as at 2022 [1]. As society faces the unavoidable process of aging, issues related to the quality of life of older adults as well as the health of older adults are attracting wider attention. Physical inactivity and chronic pain are common and serious problems in the health and lives of older adults.

Chronic pain is prevalent among older adults, with a prevalence of 25–76% among older adults in a community [2]. More than 90% of community-dwelling older adults have experienced pain in the preceding month, with 41% reporting intolerable pain [3]. Chronic pain in older adults is often complex and involves multiple diagnoses [4]. Pain regulatory mechanisms are altered in older adults, resulting in increased pain sensibility and diminished pain inhibition [5]. Chronic pain can cause significant distress, disability, and increased healthcare costs, and medication may have limited efficacy and significant side effects in older adults [6].

Chronic pain in older adults can also lead to declines in their cognitive functioning, attention, and working memory, and this is heightened by these age-related differences in pain sensibility [7]. Chronic pain faced by older adults is also commonly associated with a variety of psychological factors, such as depression and anxiety, with 20% of older adult pain feelings being heightened by these psychological issues [8].

Engaging in physical activity can help older adults manage pain, breaking the negative cycle often associated with increased discomfort [9]. Insufficient physical activity among older adults can result in a variety of detrimental health effects, such as declines in physical capabilities, a heightened likelihood of chronic illnesses, and a diminished quality of life [10]. A lack of movement can contribute significantly to systemic inflammation and muscle atrophy [11], which can worsen the health issues associated with aging. But in reality, exercise offers potential benefits for older adults with chronic pain. Moderate-intensity exercise can reduce chronic pain in older adults, although this effect may not be sustainable in the long term if participation is not sustained [12]. Regular physical activity can provide temporary pain relief, improve health, and help older adults with chronic back pain remain active [13].

The fact is that older adults face many barriers to exercise and physical activity, especially those with chronic pain. In previous studies, the common barriers included fear of falling, inertia, physical symptoms, and negative affect [14]. Chronic pain intensity can prevent people from exercising because they may feel uncomfortable and uncertain about their ability to exercise [15]. The presence of other health conditions can complicate exercise participation, leading to increased fatigue and risks associated with physical activity [16]. Environmental factors such as cost, travel time, and lack of access to exercise can also hinder participation by older adults [17]. In addition, embarrassment, the availability of certain treatments, and a lack of confidence in non-pharmacological methods can hinder pain management efforts [18]. Many older adults are also unaware of the benefits of physical activity, which can lead to a lack of motivation to participate [19]. Limited social networks can reduce motivation and opportunities to participate in group activities that are often beneficial for older adults [20].

Previous research has largely concentrated on particular types of chronic pain and ignored the preferences of older adults in terms of exercise types, settings, and the social dimensions of physical activity. Additionally, several investigations have overlooked taking into account the psychological aspects linked to exercise among older adults, such as motivation, the fear of experiencing pain, and mental health concerns.

The aim of this study was to explore the current state of pain experienced by older adults living with chronic pain in a community. It investigated not only their exercise habits and preferences but also the psychological effects of pain on their lives. Furthermore, this research sought to pinpoint the facilitating factors and barriers to physical activity, offering views into how interventions can be tailored to address both the physical and psychological needs of this population.

## 2. Methods

### 2.1. Design and Sample

This study was divided into two phases. Phase I was an exploratory cross-sectional study. Phase II involved semi-structured interviews with older adults with chronic pain. Ethical approval was obtained from the Research Ethics Committee of the Hong Kong Metropolitan University before the start of this study (reference number HE-KSPF/2024/05). The trial was also registered with the Clinical Trials Centre (registration number NCT06595810). Before the questionnaire began, participants were prompted to read the informed consent form. Once they began answering, it was assumed that they had provided their agreement to participate. The questionnaires were distributed both in the mainland Chinese communities’ civic activity centers and via the online platform “Wenjuanxing” using a snowball sampling method. Participants were recruited through referrals from initial participants, ensuring a wide reach within the target population. The inclusion criteria required participants to be aged 60 years or older and able to read and communicate in Mandarin. The exclusion criteria included being diagnosed with cognitive impairments or dementia or being unable to complete the questionnaire due to language barriers or physical limitations. The research team posted the questionnaire on “Wenjuanxing”, and completion of the questionnaire was considered consent to participate in the study. A total of 280 people met the inclusion criteria and participated in the study. In Phase II, for the telephone interviews, the participants with pain in different parts of their bodies were selected from the participants who completed the Phase I questionnaire survey. A total of 5 participants participated in the second phase of the study.

### 2.2. Procedure

Demographic data, including questions about age, gender, and chronic disease status, were collected. Psychological parameters, including quality of life, anxiety, happiness, and depression, were also collected. The exercise habits of all participants, as well as exercise frequency, exercise time, current exercise barriers, and motivation for exercise, were also collected. For the participants who reported experiencing pain in the past three months, their pain statuses were investigated, including the locations of pain, the intensity of pain, and the current use of pain relief methods.

### 2.3. Outcome Measures

#### 2.3.1. Questionnaires

A comprehensive questionnaire consisting of 58 items was crafted and divided into the following four sections: demographic data; the Chinese version of the Brief Pain Inventory (BPI-C) scale; the Depression, Anxiety, and Stress Scale (DASS-10); and barriers to exercise. In Part 1, demographic data and information on exercise habits were collected. In Part 2, the BPI-C scale was used to assess the location, prevalence, severity, and impact of pain in the last 24 h. Part 3 used the Depression, Anxiety, and Stress Scale ((DASS)-42), the recommended version of the DASS-10 scale, to assess the psychological statuses of the participants with pain. Part 4 investigated the barriers to exercise that the participants experienced during exercise.

#### 2.3.2. Brief Pain Inventory

Pain was measured using the Chinese version of the Brief Pain Inventory (BPI-C), a self-administered assessment tool that assesses pain intensity and pain interference [21]. The BPI pain intensity is the average score of 4 items asking about the worst, least severe, and average pain in the past week and the current pain intensity. The BPI pain interference measures the impact of pain on 7 life domains, including general activities, mood, ability to walk, normal work, relationships with others, sleep, and enjoyment of life. The total score ranges from 0 to 10, with higher scores indicating more severe pain [22]. This scale demonstrates good reliability, and the Cronbach’s alpha coefficients were 0.85 and 0.88 for the intensity items, respectively [23].

#### 2.3.3. Depression, Anxiety, and Stress Scale (Dass-10)

The psychological statuses of the elderly participants were measured using the brief version of the Depression, Anxiety, and Stress Scale (DASS-10) [24], which is a 10-item version of the widely used Depression, Anxiety, and Stress (DASS)-42 scale, aiming for it to be easily accessed. This scale contains a total of 10 questions to assess changes in depression, anxiety, and stress levels in the elderly, with scores ranging from 0 to 3, and increasing scores mean increased levels of depression, anxiety, and stress. It shows high internal reliability, with a Cronbach’s alpha of 0.89 [25].

#### 2.3.4. Validity Test and Test–Retest Reliability

Four experts in the field of pain management were invited to participate in the review process, including a registered nurse, two occupational therapists, and a Chinese medicine expert. The item levels of the content validity index (I-CVI) for each item in the questionnaire ranged from 0.9 to 1.0, all of which were higher than 0.78.

### 2.4. Data Analysis

Statistical Package for the Social Sciences (SPSS) 27.0 was used to analyze the data. Descriptive statistics were employed to examine the quantitative data. To examine the differences in demographic outcomes as well as psychological impacts between those with and without pain, chi-square tests and independent *t*-tests were used. A *p*-value of <0.05 was considered statistically significant.

Semi-structured interviews were used to gather qualitative insights, and subsequently, the data obtained from these interviews were analyzed. Semi-structured interviews serve as a valuable qualitative research method designed to extract information on a particular subject or field based on individual experiences [26]. In this study, data were collected from 5 older participants regarding their experiences with pain and their exercise routines.

## 3. Results

### 3.1. Demographic Data

There were 280 participants (143 males and 137 females, mean age = 65.88 years) who joined the study. A majority of the patients (83.21%) suffered from chronic conditions, with hypertension (59.64%) and arthritis (39.32%) being the most common. Demographic characteristics such as gender, age, marital status, education, occupation, residential status, and income status were compared between the participants in the pain group and those in the no pain group. Other crucial variables were also assessed, such as the individuals’ chronic illness status. It was found that 187 (66.79%) participants had experienced pain in the past 3 months, and they were considered the pain group, whereas 93 (33.21%) had not felt pain in the past 3 months and were considered the no pain group. The demographics of the participants with and without pain differed significantly by gender, and several chronic conditions also showed significant differences between the two groups, including hypertension, diabetes, arthritis, and cataracts (Table 1).

### 3.2. Exercise Habits and Preferences

All participants were asked about their exercise habits as well as preferences. Less than half of the participants (43.32%) exercised for more than 30 min at a time. The participants without pain (64.52%) exercised significantly more often than three times per week compared to the participants with pain (48.66) (*p* = 0.012). Of all the exercises, the most frequently selected exercise was walking (69.52%), followed by jogging (42.78%) and table tennis (27.27%). The participants without pain significantly preferred walking (*p* = 0.017) and table tennis (*p* = 0.002) (Table 2).

### 3.3. Pain Situations: Pain Sites and Pain Intensities

**Figure 1:**
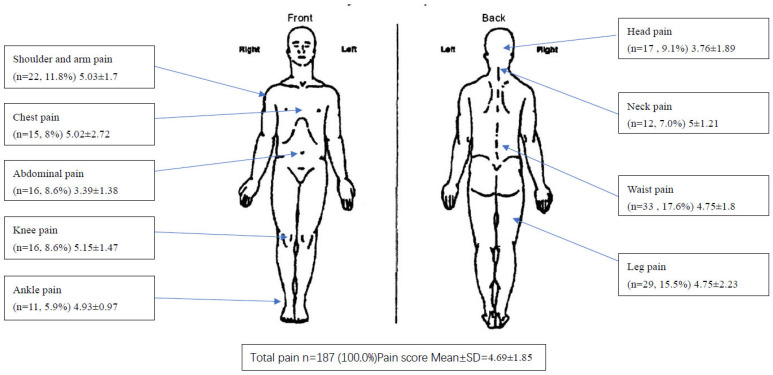
Pain sites and pain intensities.

Approximately 66.79% (*n* = 187) of the participants had suffered pain in the past 3 months, with the most common sites being the lower back (17.6%, with a pain score of 4.75), legs (15.5%, with a pain score of 4.75), shoulders as well as arms (11.8%, with a pain score of 5.03), and head (9.1%, with a pain score of 3.76). The overall mean pain intensity in the pain group was 4.69 ± 1.85 (Figure 1).

### 3.4. Interference of Pain in Daily Life

Fifty-four-point fifty-five percent of participants (*n* = 102) with pain experienced a mild impact of pain on various facets of their lives, while 29.41% reported a moderate impact and 16.04% reported a severe impact. A majority of the participants reported a mild impact of their pain, and as the intensity of their pain increased, so did the impact on various facets of their lives. The participants who reported severe impacts on their lives due to pain experienced significantly higher pain levels compared to those with mild or moderate impacts (*p* < 0.001) (Table 3).

### 3.5. Psychological Status and Relationship with Pain

Table 4 shows the disparity in psychological status (measured by the DASS-10) among those experiencing pain versus those without it. The pain group exhibited a mean DASS-10 score of 2.6667 ± 2.0495 for mild distress, while the non-pain group scored 2.9737 ± 2.0975. For moderate distress, the pain group averaged 8.8478 ± 1.8274, compared to 9.7 ± 2.0527 for the non-pain group. In terms of severe distress, the pain group’s mean was 19.1296 ± 3.638, whereas the non-pain group’s mean was 18.76 ± 3.1723. Different pain states had impacts on the psychological states of the different participants (*p* < 0.001). The DASS-10 scores for mild and severe psychological distress in the group without pain were lower than those in the group with pain. Conversely, in the moderate distress group, individuals without pain exhibited higher scores than those with pain.

### 3.6. Facilitation Factors for Exercise

The most important motivator for all participants to exercise was support from family and friends (73.21%), followed by the availability of sports facilities (61.43%) and professional advice. The most significant differences between the participants with and without pain regarding motivation to exercise were that the participants without pain preferred community exercise programs (*p* = 0.001) and received more advice from experts (*p* = 0.045). The strategies to enhance physical activity that were preferred by all participants included increasing physical activity companionship (*n* = 211, 75.36%), followed by reducing barriers to exercise and increasing the facilities where they exercised (54.29%). The environment in which the exercise took place was also equally important in increasing participants’ physical activity levels (Table 5).

### 3.7. Barriers to Exercise

A majority (*n* = 168, 60%) of the participants reported that they refrained from engaging in physical exercise primarily due to the experience of pain (*p* = 0.002), indicating a strong correlation between pain and exercise avoidance; however, it is noteworthy that the predominant factor contributing to the lack of exercise among the participants was attributed to an overall absence of motivation (61.07%). This suggests that while pain may be a significant barrier to physical activity, motivational factors play a more substantial role in shaping exercise behavior (Table 5).

### 3.8. Qualitative Findings on Older Adults’ Pain and Exercise Levels

A qualitative analysis of the participants’ experiences with barriers and facilities for exercise revealed a complex set of factors that, together, constituted a deep-seated dilemma constraining exercise participation among these older adults. Chronic diseases, particularly physical ailments such as neuralgia and gout, often deter older adults from considering increased exercise. To make matters even more difficult, many older adults harbor an instinctive resistance to exercise that stems not only from anxiety about the possibility of accidents but also from an unspeakable resistance to physical activity itself. To add insult to injury, the high costs of gym memberships and medical counseling add another financial hurdle to already hesitant older adults.

The participants also mentioned that the experience of exercise with friends and family not only made exercise fun but also provided a great deal of comfort on a spiritual level, as the positive effects of such group interactions can often bring a different luster to boring sports activities. Improvements in community infrastructure, such as better neighborhood conditions, were also noted as encouraging more walking and outdoor activities (Table 6).

## 4. Discussion

The incidence of chronic pain among the participants in this study was quite high. Out of 280 participants, approximately 66.8% of the participants were experiencing pain, with an average pain score of 4.69 on a scale of 1 to 10. The most common pain sites were the lower back and legs, and for both areas, the participants reported with high percentages. Anxiety and stress levels were significantly higher among those in the pain group.

This study indicates the profound influence that chronic pain exerts on the exercise habits and psychological well-being of older adults. Chronic pain often leads to a reduction in physical activity, which, in turn, can exacerbate feelings of depression, anxiety, and social isolation among older adults. The interrelationship between chronic pain and physical inactivity is multifaceted; individuals experiencing persistent pain may find it increasingly difficult to engage in regular exercise, which is essential for maintaining not only physical health but also mental well-being.

The demographics of the pain and no-pain groups differed only by gender, with no notable distinctions in other attributes. Over three-quarters of the study participants (83.21%) grappled with chronic conditions, with hypertension (59.64%) and arthritis (39.32%) topping the list. These findings aligned with earlier research on chronic conditions in older Chinese adults [27]. When it came to fitness routines and inclinations, fewer than half of the participants (43.32%) engaged in prolonged workouts lasting over 30 min. Members of the pain-free group were more active, exercising over three times a week, a significantly higher frequency than those in the pain group. Pain clearly diminishes the exercise frequency among older adults, illustrating that whether or not they experience pain is a major determinant of their physical activity levels [28]. Chronic pain significantly reduces exercise frequency among older adults, with pain-free older adults being more active.

Approximately 66.79% of the participants reported pain in the last three months, with a mean pain intensity of 4.69 in the pain group. Common pain locations included the lower back, legs, shoulders, and arms. This aligned with previous studies, highlighting chronic lower back pain and osteoarthritis-related discomfort in the legs and hips in older adults [29,30]. Furthermore, it resonated with another study, noting a connection between activity levels and shoulder pain, along with the prevalence of shoulder and hand pain [31].

Pain significantly affects daily life, with 54.55% of the participants noting a mild impact, 29.41% a moderate impact, and 16.04% a severe impact. It impairs physical functioning and overall quality of life, diminishing well-being across various domains [32]. Similar findings have indicated that pain disrupts daily activities, mood, and sleep [33]. Moreover, the type of pain may influence specific life activities; for instance, neck pain correlates with reduced mobility and sleep issues [34]. Consequently, older adults with lower back, leg, and shoulder pain experience decreased physical activity.

Participants in the pain group showed higher levels of anxiety, depression, and stress compared to the no-pain group. The higher the level of perceived pain disturbance, the higher the probability of reporting less favorable mental health status [35]. Over 25% of the participants in both groups experienced severe distress, suggesting that the distress levels in both groups were high despite differences in pain status. For the non-pain group, distress may have been influenced by other sources of psychological stress, such as social isolation, financial issues, or other health problems [36]. One particularly noteworthy aspect of this study was the finding that, while both the mild and severe distress scores were higher in the pain group, moderate distress was less pronounced in this group. This suggests that individuals with chronic pain may develop certain coping mechanisms, such as acceptance or habituation, that help manage distress at moderate levels [37]. This coping response may help explain the lower scores observed in the pain group for moderate distress compared to the non-pain group, whose responses to moderate stressors may have been more emotionally reactive. This also demonstrates the need for better pain management strategies for older adults as well as pain management approaches that promote their mental health [38]. The complex psychological experience of pain, often intertwined with physical pain, forms an indivisible whole. Regarding the feeling of pain in older adults, we have to think about a more macroscopic perspective—those seemingly independent negative emotions may actually originate from the superposition of multidimensional mental factors and personal experiences.

The most significant facilitator for exercise participation was the support from family and friends. This was aligned with a previous study where family and friends increased motivation and accountability, making it easier for older adults to maintain an active lifestyle [33]. The availability of exercise facilities and the cost of these facilities were also noted as important factors. Participants without pain showed a stronger preference for community exercise programs and expert-guided exercises, as knowledgeable experts can increase motivation and adherence to exercise routines [39], indicating that professional guidance can significantly enhance exercise participation in individuals.

The barriers to exercise remain significant. The primary reason for avoiding physical activity is pain, which becomes an excuse for people to avoid exercise, creating a vicious cycle that is difficult to break. This is particularly true for the older adults, where excessive concerns about their existing pain often become a barrier to exercise [40]. But the most substantial barrier was the overall lack of motivation. There is a general lack of knowledge about the benefits of exercise [41], and motivating variables have a greater influence on exercise behavior.

The qualitative findings revealed that in exercising with friends and family, the participants not only experienced joy during exercise but also received comfort and support on a spiritual level. The social factor of group interaction undoubtedly brings a different kind of vitality and charm to boring sports activities. The choice of exercise environment is also crucial, showing the urgency of creating safe and convenient exercise space for older adults. Unfortunately, the high costs of gym memberships and medical counseling add a financial burden, and this dilemma largely restricts older adults’ motivation to engage in sports activities.

The study’s limitations included the limited sample size in both the qualitative and quantitative parts and the limited demographic diversity, which may have affected the generalizability of the findings to a broader population. There were 280 participants included in the study, but the data were mainly derived from a specific community, which makes it difficult to fully reflect the true picture of chronic pain among older adults in different geographic, economic, and cultural contexts. Future research should include a larger and more diverse sample to improve the external validity of the results. Also, data on pain experience and exercise habits collected based on self-reports are often subject to subjective bias, resulting in the distorted reporting of symptoms and activity intensity. To enhance reliability, objective assessment tools such as smartphone exercise apps should be introduced in subsequent studies and cross-validated with self-reported data, with a view to obtaining more reliable findings. Future research should incorporate a longitudinal approach to evaluate the long-term effects of chronic pain on exercise habits and mental health, which would provide a more comprehensive understanding of how these factors evolve over time.

## 5. Conclusions

In this survey, 280 participants joined our study, and 66.8% of them suffered from pain of a relatively high intensity. Promoting physical activity can reduce chronic pain, which highlights the necessity of exploring exercise habits in older adults with chronic pain. We found that pain discouraged physical activity, while active individuals were more engaged in enjoyable exercises like walking and table tennis. Given the prevalence of chronic diseases and chronic pain, a comprehensive approach is essential to address both the physical and psychological challenges faced by these individuals to promote physical activity. The key barriers identified included pain, mental health issues, and financial constraints, while social support and environmental improvements served as strong motivators. In future research, we may be able to help older adults in multiple physical, psychological, and social aspects, such as adding peer and intergenerational support as well as a technology-based e-coaching exercise guide to increase their physical activity and release chronic pain.

## Figures and Tables

**Table 1:** Demographic data (*n* = 280).

	Total (*n* = 280)	Pain Group (*n* = 187, 66.79%)	No Pain Group (*n* = 93, 33.21%)	*t*-Test	
	*n* (%)	*n* (%)	*n* (%)	*p*-Value α	*p*-Value β
**Age** α	65.88 ± 4.515	65.46 ± 4.238	68.65 ± 5.308	0.077	
**Gender**					0.008 *
Male	143 (51.1)	127 (52.3)	16 (43.2)		
Female	137 (48.9)	116 (47.7)	21 (56.8)		
**Marriage**					0.216
Unmarried	2 (0.71)	2 (0.82)	0 (0.00)		
Married	208 (74.29)	188 (77.37)	20 (54.05)		
Divorced	30 (10.71)	21 (8.64)	9 (24.32)		
Widowed	38 (13.57)	31 (12.76)	7 (18.92)		
Other	2 (0.71)	1 (0.41)	1 (2.7)		
**Education**					
No education	18 (6.43)	10 (5.35)	10 (5.35)		0.507
Elementary school	87 (31.07)	62 (26.88)	62 (33.16)		
Secondary school	105 (37.5)	71 (36.56)	71 (37.97)		
College and above	70 (25.00)	44 (27.96)	44 (23.53)		
**Occupation**					0.059
Full-time Job	23 (8.21)	11 (12.9)	11 (5.88)		
Part-time Job	11 (3.93)	10 (1.08)	10 (5.35)		
Unemployed	54 (19.29)	40 (15.05)	40 (21.39)		
Retirement	190 (67.86)	124 (70.97)	124 (66.31)		
Oher	2 (0.71)	2 (0)	2 (1.07)		
**Income**					0.155
No income	34 (12.14)	28 (6.45)	28 (14.97)		
<10,000 a month	167 (59.64)	110 (61.29)	110 (58.82)		
>10,000 a month	74 (26.43)	47 (29.03)	47 (25.13)		
Unstable	1 (0.36)	0 (1.08)	0 (0)		
Refuse to answer	4 (1.43)	2 (1.07)	2 (1.07)		
**Living conditions**					0.077
Living alone	31 (11.07)	16 (8.56)	15 (16.13)		
Living with spouse	145 (51.79)	93 (49.73)	52 (55.91)		
Living with children	85 (30.36)	64 (34.22)	21 (22.58)		
Living with parents	19 (6.79)	14 (7.49)	5 (5.38)		
**Chronic diseases**					
No diseases	47 (16.79)	18 (9.63)	29 (31.18)		<0.001 **
Hypertension	167 (59.64)	121 (64.71)	46 (49.46)		0.014 *
Diabetes	90 (32.14)	70 (37.43)	20 (21.51)		0.007 *
Heart disease	64 (22.86)	49 (26.2)	15 (16.13)		0.059
Stroke	21 (7.5)	16 (8.56)	5 (5.38)		0.341
Tracheal disease	50 (17.86)	39 (20.86)	11 (11.83)		0.063
Arthritis	110 (39.29)	82 (43.85)	28 (30.11)		0.027 *
Cataracts	35 (12.5)	29 (15.51)	6 (6.45)		0.031 *
Cancer	15 (5.36)	10 (5.35)	5 (5.38)		0.992

**α: an independent *t*-test was used. β: a chi-squared test was used**. **, *p* < 0.001 and *, *p* < 0.05, which were considered statistically significant.

**Table 2:** Exercise habits and preferences.

	Total (*n* = 280)	Pain Group (*n* = 187, 66.79%)	No Pain Group (*n* = 93, 33.21%)	
	*n* (%)	*n* (%)	*n* (%)	*p*-Value
**Exercise habit**				0.629
Yes	243 (86.1)	161 (86.1)	82 (88.17)	
No	37 (13.9)	26 (13.9)	11 (11.83)	
**Exercise frequency**				0.012 *
<3 times a week	129 (51.34)	96 (51.34)	33 (35.48)	
≥3 times a week	151 (48.66)	91 (48.66)	60 (64.52)	
**Exercise time**				0.423
<30 min at a time	154 (56.68)	106 (56.68)	48 (51.61)	
≥30 min at a time	126 (43.32)	81 (43.32)	45 (48.39)	
**Types of exercise**				
Walking	207 (69.52)	130 (69.52)	77 (82.8)	0.017 *
Jogging	126 (42.78)	80 (42.78)	46 (49.46)	0.290
Bicycling	63 (24.06)	45 (24.06)	18 (19.35)	0.374
Swimming	55 (19.79)	37 (19.79)	18 (19.35)	0.932
Yoga	39 (16.04)	30 (16.04)	9 (9.68)	0.147
Gym training	56 (19.79)	37 (19.79)	19 (20.43)	0.899
Ping pong	94 (27.27)	51 (27.27)	43 (46.24)	0.002 *
Basketball	32 (9.63)	18 (9.63)	14 (15.05)	0.179
Soccer	54 (18.72)	35 (18.72)	19 (20.43)	0.732
Handball	30 (11.23)	21 (11.23)	9 (9.68)	0.692

**A chi-squared test was used.** *, *p* < 0.05, which was considered statistically significant.

**Table 3:** Pain situation.

	Pain (*n* = 187)				
	*n* (%)	Mean	SD	F	*p*-Value
**Impact on life**				39.072	<0.001 *
Mild affect	102 (54.55)	3.7794	1.56315		
Moderate affect	55 (29.41)	5.6364	1.34010		
Severe affect	30 (16.04)	6.0667	1.90500		

**One-way Anova was used.** *, *p* < 0.05, which was considered statistically significant.

**Table 4:** Dass-10.

	Total (*n* = 280)	Pain Group (*n* = 187, 66.79%)			No Pain Group (*n* = 93, 33.21%)		
	*n* (%)	Mean ± SD	*n* (%)	Mean ± SD	*n* (%)	Mean ± SD	*p*-Value
**Dass-10**							<0.001 **
Mild distress	125 (44.64)	2.76 ± 2.0648	87 (46.52)	2.6667 ± 2.0495	38 (40.86)	2.9737 ± 2.0975	
Moderate distress	76 (27.14)	9.1842 ± 1.9579	46 (24.6)	8.8478 ± 1.8274	30 (32.26)	9.7 ± 2.0527	
Severe distress	79 (28.21)	19.0127 ± 3.4916	54 (28.88)	19.1296 ± 3.638	25 (26.88)	18.76 ± 3.1723	

**Compare means and Anova tests were used.** **, *p* < 0.001 was considered statistically significant.

**Table 5:** Barriers and motivations for exercise.

	Total (*n* = 280)	Pain Group (*n* = 187, 66.79%)	No Pain Group (*n* = 93, 33.21%)	
	*n* (%)	*n* (%)	*n* (%)	*p*-Value
**Motivation for exercise**				
Professional advice	154 (55)	95 (50.8)	59 (63.44)	0.045 *
Familial and friend support	205 (73.21)	132 (70.59)	73 (78.49)	0.159
Sports facilities	172 (61.43)	111 (59.36)	61 (65.59)	0.313
Weather and seasons	133 (47.5)	95 (50.8)	38 (40.86)	0.117
Community sports activities	99 (35.36)	54 (28.88)	45 (48.39)	0.001 *
**Reasons for discontinuing the exercise**				
Pain	168 (60)	124 (66.31)	44 (47.31)	0.002 *
Lack of time	153 (54.64)	97 (51.87)	56 (60.22)	0.187
Lack of motivation	171 (61.07)	110 (58.82)	61 (65.59)	0.274
Environmental	110 (39.29)	68 (36.36)	42 (45.16)	0.156
**Strategies for increasing exercise**				
Provision of sports facilities	152 (54.29)	93 (49.73)	59 (63.44)	0.03 *
Sports benefits education	189 (67.5)	123 (65.78)	66 (70.97)	0.382
Increase companionship	211 (75.36)	138 (73.8)	73 (78.49)	0.39
Reducing barriers to exercise	152 (54.29)	101 (54.01)	51 (54.84)	0.896

**A chi-squared test was used.** *, *p* < 0.05, which were considered statistically significant.

**Table 6:** Qualitative analysis of participant feedback on exercise barriers and benefits.

Survey Query	Feedback from Participants	Themes
Exercise barriers experienced in life	Health issues I have nerve pain in my back and legs and do not want to do extra exercise at all. “A gout attack makes the ankles so difficult to stand, not to mention exercise.”	Health issues Psychological barriers Expenses
	Psychological barriers “Currently getting enough exercise and don’t want to exercise unless multiple doctors ask for it” “Of course I know exercise is important, but I still worry about injuries from inappropriate exercise.” “Do not like to exercise and do not have time for extra exercise”	
	Expenses “When I couldn’t stop the pain in my knee, I went to the hospital to learn what to do to relieve it, and for a couple hundred bucks I was taught just a few small movements.” “I wish I could move more, but the gym isn’t free.”	
Advantages of exercise	Company “Often go out hiking with friends, and the park is free for older adults. You can also hang out with friends and gossip.” “Sharing information about exercise so my daughter won’t worry about my health.”	Company Environment
	Environment “The neighborhood is great, and I’ve been walking more since the environmental repairs and improvements were made”	

## Data Availability

The datasets presented in this article are not readily available because the data are part of an ongoing study. Requests to access the datasets should be directed to mmytse@hkmu.edu.hk.
